# Nutrition-Related Indices and Systemic Inflammation in Acute Coronary Syndrome: Prognostic Utility of PNI with IPI/AISI and Links to Angiographic Severity and Survival

**DOI:** 10.3390/nu18060971

**Published:** 2026-03-19

**Authors:** Nedim Uzun, Naile Fevziye Misirlioglu, Seyma Dumur, Sinem Durmus, Aysun Ekinci, Hafize Uzun

**Affiliations:** 1 Department of Emergency, Gaziosmanpaşa Training and Research Hospital, University of Health Sciences, Istanbul 34250, Türkiye; 2 Department of Medical Biochemistry, Faculty of Medicine, Istanbul Atlas University, Istanbul 34403, Türkiye; nailemisirlioglu@gmail.com (N.F.M.); seyma.dumur@atlas.edu.tr (S.D.); huzun59@hotmail.com (H.U.); 3 Department of Medical Biochemistry, Faculty of Medicine, Izmir Katip Çelebi University, İzmir 35620, Türkiye; durmus.sinem@gmail.com; 4 Department of Medical Biochemistry, Faculty of Medicine, Dicle University, Diyarbakir 21280, Türkiye; draysunekinci@gmail.com

**Keywords:** NSTEMI, STEMI, prognostic nutritional index, inflammatory prognostic index, aggregate index of systemic inflammation, Gensini score, survival, Cox regression

## Abstract

**Background:** Acute coronary syndrome (ACS) remains a leading cause of morbidity and mortality worldwide, and improved risk stratification beyond conventional biomarkers is needed. Novel laboratory-derived indices reflecting systemic inflammation and immunonutritional status including the inflammatory prognostic index (IPI), prognostic nutritional index (PNI), and aggregate index of systemic inflammation (AISI) may provide integrated prognostic information in ACS. **Methods:** In this cohort study, 2400 participants were included: 800 controls, 800 patients with non-ST-elevation myocardial infarction (NSTEMI), and 800 with ST-elevation myocardial infarction (STEMI). **Results:** Compared with controls, NSTEMI and STEMI patients were younger and exhibited higher body mass index, blood pressure, heart rate, and progressively worse glycemic indices (fasting glucose and HbA1c; all *p* < 0.001). Lipid parameters were significantly higher in ACS groups versus controls (*p* < 0.001). Cardiac biomarkers were markedly elevated in ACS, with significantly higher troponin I and CK-MB levels in STEMI than NSTEMI and controls (both *p* < 0.001). Inflammatory and renal parameters (CRP, fibrinogen, urea, creatinine) were increased in ACS, most prominently in STEMI. Composite indices demonstrated strong inter-correlations, including a strong positive correlation between AISI and IPI (r ≈ 0.91, *p* < 0.001), while PNI correlated inversely with CONUT score (r ≈ −0.70, *p* < 0.001). The Gensini score differed significantly among groups and was highest in NSTEMI (*p* < 0.001). Survival was significantly lower in STEMI than NSTEMI (log-rank *p* = 0.005), with RMST of 315.5 days in NSTEMI versus 299.4 days in STEMI. In multivariable Cox regression, STEMI presentation independently predicted higher mortality risk (HR 1.26, 95% CI 1.04–1.53; *p* = 0.018), and higher Gensini score was also independently associated with mortality (HR 1.01 per point; 95% CI 1.00–1.02; *p* = 0.036). Higher PNI was independently protective (HR 0.997; 95% CI 0.993–1.000; *p* = 0.045), whereas age and CONUT score were not significant in the adjusted model. **Conclusions:** Novel laboratory-derived systemic inflammatory and nutrition-related indices particularly IPI and AISI as markers of inflammatory burden and PNI as a marker of immunonutritional balance provide clinically relevant prognostic information in ACS. STEMI presentation is associated with shorter survival, and all-cause mortality is independently related to STEMI status, greater angiographic severity (higher Gensini score), and lower PNI. These readily available indices may offer incremental value for risk stratification in NSTEMI and STEMI when integrated with conventional clinical and angiographic assessment.

## 1. Introduction

Acute coronary syndrome (ACS) encompasses a high-risk clinical spectrum, including ST-elevation myocardial infarction (STEMI), non-ST-elevation myocardial infarction (NSTEMI), and unstable angina. Representing the symptomatic manifestation of coronary heart disease, ACS significantly contributes to global mortality, accounting for approximately one-third of deaths in the population aged over 35 [1,2,3]. Unlike other potentially silent coronary pathologies, ACS is characterized by acute symptomatic presentations, necessitating rapid risk stratification and clinical intervention [2].

Despite revolutionary advances in percutaneous coronary intervention (PCI) and pharmacotherapy, risk stratification in the acute phase continues to be a challenge [4]. Current clinical practice relies heavily on conventional cardiac biomarkers, such as troponins, and traditional risk scores. However, these parameters often fail to capture the complex interplay between systemic inflammation and nutritional status, both of which are critical determinants of atherosclerotic plaque instability and post-ischemic recovery [5].

Emerging evidence suggests that atherosclerosis is not merely a lipid-sequestration disease but a chronic inflammatory process [6]. Recent research has focused on composite indices derived from routine complete blood counts and biochemical markers, which offer a more comprehensive reflection of the systemic inflammatory milieu [6,7,8,9,10]. The inflammatory prognostic index (IPI) and the aggregate index of systemic inflammation (AISI) have recently gained attention for their ability to integrate multiple inflammatory pathways [11,12,13,14]. Furthermore, the prognostic nutritional index (PNI) and the controlling nutritional status (CONUT) score have been identified as vital indicators of the host’s nutritional–inflammatory balance, which is often compromised in the setting of acute myocardial injury [15,16,17,18].

While the individual prognostic value of certain inflammatory cells is well-documented, there is a scarcity of data directly comparing the integrative performance of these novel indices (IPI, AISI, PNI) across the full spectrum of ACS [11,12,13,14]. Moreover, the correlation between these systemic markers and the anatomical severity of coronary artery disease (CAD), quantified by the Gensini score [19,20], remains insufficiently explored in large-scale comparative cohorts of STEMI and NSTEMI patients [20].

This study aimed to evaluate the prognostic utility of novel systemic inflammatory and nutrition-related indices in patients presenting with ACS. Specifically, we (i) compared these indices across STEMI, NSTEMI, and non-ACS control groups; (ii) examined their associations with angiographic disease severity using the Gensini score; and (iii) assessed their independent predictive value for all-cause mortality. We hypothesized that these readily available, laboratory-derived integrative indices would enhance risk stratification beyond conventional parameters and may provide a practical, cost-effective adjunct to routine clinical assessment in ACS.

## 2. Materials and Methods

### 2.1. Study Design and Setting

This retrospective cohort study was conducted at Gaziosmanpaşa Training and Research Hospital, a tertiary-care referral center located in Istanbul, Türkiye, and included consecutive adult participants evaluated for ACS during the study period. The study population was categorized into three groups: (i) patients presenting with ST-elevation myocardial infarction (STEMI), (ii) patients with non-ST-elevation myocardial infarction (NSTEMI), and (iii) control subjects without ACS.

#### 2.1.1. Ethical Approval

The study was performed in accordance with the Declaration of Helsinki. Ethical approval was granted by the Clinical Research Ethics Committee of Gaziosmanpasa Training and Research Hospital (Approval No: 180; Date: 7 January 2026). Given the retrospective design and use of de-identified routine clinical data, informed consent requirements were managed according to institutional ethics committee regulations.

#### 2.1.2. Study Population

##### ACS Groups (NSTEMI and STEMI)

A total of 1600 ACS patients (800 NSTEMI and 800 STEMI) were included. Patients were eligible if they:

1. Were ≥18 years of age;
2. Had a final diagnosis of STEMI or NSTEMI during index hospitalization;
3. Underwent coronary angiography during acute admission or within the clinically indicated time window.

#### 2.1.3. Diagnostic Criteria

Patients were included based on the diagnostic criteria for NSTEMI and STEMI according to ESC/ACC guidelines [21]. Patients with ST elevation or new left bundle branch block on ECG were included in the STEMI group. ACS diagnoses were established according to contemporary guideline-based definitions at the time of care, integrating symptoms, electrocardiography, and cardiac biomarkers. STEMI was defined by persistent ST-segment elevation (or new/presumed new left bundle branch block when clinically appropriate) with evidence of myocardial necrosis. NSTEMI was defined by a rise and/or fall in cardiac troponin with at least one value above the 99th percentile upper reference limit in the absence of persistent ST-segment elevation, accompanied by ischemic symptoms and/or ischemic ECG changes.

#### 2.1.4. Control Group

The control group comprised 800 adults without ACS who were evaluated at the same institution during the same study period. Controls were selected from individuals undergoing routine cardiovascular assessment (primarily in the outpatient setting) who had no evidence of an acute ischemic event at presentation, no clinical diagnosis of myocardial infarction (STEMI/NSTEMI), and no acute rise/fall pattern in cardiac biomarkers. Baseline laboratory measurements were obtained at the time of index evaluation using the same institutional protocols and analyzers as in the ACS groups and were available for all variables required to calculate the studied indices (PNI, IPI, AISI, and CONUT). Coronary angiography was not required for inclusion and was not routinely performed in controls; accordingly, Gensini scoring was not assessed in the control group. Therefore, the control cohort represents a non-ACS comparator group and may include individuals with stable coronary artery disease or cardiovascular risk factors.

#### 2.1.5. Exclusion Criteria

Participants were excluded if they had any of the following: active infection or sepsis at presentation; chronic inflammatory/autoimmune disease with active flare or immunosuppressive therapy; hematologic malignancy or active solid malignancy; severe hepatic failure; end-stage renal disease requiring dialysis; recent major surgery or trauma (e.g., within 4–6 weeks); incomplete key laboratory data necessary to calculate IPI/PNI/AISI/CONUT; or missing follow-up data for mortality analyses.

#### 2.1.6. Data Collection

Data were extracted from electronic medical records and included demographic characteristics (age, sex), anthropometric measures (body mass index, BMI), vital signs at admission (systolic and diastolic blood pressure, heart rate), documented medical history and comorbidities, laboratory parameters obtained at admission or from the first available sample prior to invasive procedures, coronary angiography findings including Gensini score (when available), and clinical outcomes (all-cause mortality and follow-up duration).

Comorbidities were defined according to established clinical criteria. Diabetes mellitus was defined as a documented prior diagnosis, use of glucose-lowering medication, fasting plasma glucose ≥ 126 mg/dL, or HbA1c ≥ 6.5%. Hypertension was defined as a documented diagnosis, use of antihypertensive medication, or blood pressure ≥ 140/90 mmHg on repeated measurements. Dyslipidemia was defined as a documented diagnosis, use of lipid-lowering therapy, or lipid levels exceeding guideline-recommended thresholds. Smoking status was classified as current, former, or never smoker based on medical records. Obesity was defined as BMI ≥ 30 kg/m^2^. Impaired glycemic control was defined as fasting glucose and/or HbA1c levels above established diagnostic cut-offs.

### 2.2. Blood Sampling and Laboratory Measurements

#### 2.2.1. Sample Collection

Peripheral venous blood samples were obtained as part of routine clinical care at the time of initial presentation. In the ACS groups, sampling was performed upon arrival to the emergency department or immediately after admission, typically within the first hours of evaluation and prior to the initiation of intravenous fluid resuscitation and before coronary angiography/PCI whenever feasible, to minimize procedural and hemodilution-related effects. In the control group, samples were collected at the time of index clinical evaluation using the same institutional protocols. Complete blood count (CBC) parameters—including total white blood cell count, neutrophils, lymphocytes, monocytes, platelets, hemoglobin, and related indices—were measured using standardized hospital hematology analyzers operating under routine internal quality control procedures. CBC results were generated using an automated hematology analyzer (Sysmex XN-1000; Sysmex, Norderstedt, Germany).

#### 2.2.2. Biochemistry and Cardiac Biomarkers

Routine biochemical measurements were performed in the hospital’s central laboratory using enzymatic assay techniques on an automated COBAS 8000 clinical chemistry system (Roche Diagnostics, Tokyo, Japan). Assessed parameters included fasting glucose, HbA1c, lipid profile (total cholesterol, LDL cholesterol, triglycerides), renal function markers (urea, creatinine), inflammatory markers (C-reactive protein, CRP; fibrinogen), and nutrition-related markers (albumin and total protein). Cardiac biomarkers (troponin I and CK-MB) were analyzed using standardized immunoassay modules integrated within the same platform or affiliated systems according to routine clinical protocols. The laboratory operates under national accreditation standards and adheres to established internal and external quality control procedures. Daily internal quality controls, routine calibration, and periodic external proficiency testing were conducted in accordance with manufacturer recommendations, and analytical performance parameters were maintained within predefined acceptable quality limits throughout the study period.

#### 2.2.3. Definition and Calculation of Inflammatory and Nutritional Indices

All composite indices were calculated using laboratory values obtained at baseline (index admission), as follows:

Aggregate Index of Systemic Inflammation (AISI) [12,13]:

$$\mathrm{AISI}=\frac{\mathrm{Neutrophils}\times \mathrm{Monocytes}\times \mathrm{Platelets}}{\mathrm{Lymphocytes}}$$

Prognostic Nutritional Index (PNI) [15]:

$$\mathrm{PNI}=10\times \mathrm{Serum}\;\mathrm{albumin}\;(g/\mathrm{dL})+0.005\times {\mathrm{Total}\;\mathrm{lymphocyte}\;\mathrm{count}\;(/\mathrm{mm}}^{3})$$

Inflammatory Prognostic Index (IPI) [14]:

IPI = CRP × NLR/Serum albumin

CONUT score [15]:

CONUT score = Serum albumin score + Total lymphocyte count score + Total cholesterol score.

In CONUT scoring, total lymphocyte count, serum albumin amount and serum total cholesterol count were used to score between 0 and 12 points. A score of 1 was recorded as normally fed, 2–4 as mild, 5–8 as moderate and 9 and above as severe malnutrition (Table 1) [15].

### 2.3. Coronary Angiography and Gensini Score

Coronary angiography was performed using standard Judkins technique via radial or femoral access at the discretion of the interventional cardiologist [21]. Angiographic images were reviewed by experienced cardiologists blinded to the calculated inflammatory/nutritional indices when feasible for retrospective scoring. CAD severity was quantified using the Gensini score, which grades stenosis severity (e.g., 1–32 scale depending on percentage narrowing) and applies a multiplier based on lesion location and functional significance within the coronary tree. The total Gensini score was calculated as the sum of weighted scores across all lesions.

#### 2.3.1. Follow-Up and Outcomes

##### Primary Outcome

The primary endpoint was all-cause mortality during follow-up.

##### Survival Analyses

Time-to-event was defined as the interval from index admission (or diagnosis date) to death or last known follow-up. Survival comparisons between STEMI and NSTEMI were performed using Kaplan–Meier curves and the log-rank test. In addition, restricted mean survival time (RMST) was estimated over the prespecified follow-up horizon to provide an absolute measure of mean survival time.

### 2.4. Statistical Analysis

Statistical analyses were performed using JASP software (version 0.19.3; JASP Team, Amsterdam, The Netherlands). Continuous variables were assessed for normality using visual inspection of histograms and Q–Q plots. Normally distributed continuous variables are presented as mean ± standard deviation. Comparisons among the three study groups (control, NSTEMI, and STEMI) were performed using one-way analysis of variance (ANOVA) for continuous variables. When a significant overall difference was detected, post hoc pairwise comparisons were conducted using the Tukey honestly significant difference (HSD) test. Categorical variables were compared using the chi-square test, as appropriate. Survival analyses were performed after excluding the control group due to the absence of follow-up data. Kaplan–Meier survival curves were constructed to compare survival between NSTEMI and STEMI patients, and differences between groups were assessed using the log-rank (Mantel–Haenszel) test. Because median survival time was not reached during follow-up, RMST was calculated to summarize survival differences between groups. For the Cox proportional hazards model, all clinically relevant variables were first examined in univariable analyses. Specifically, age, sex, body mass index (BMI), diabetes mellitus, hypertension, dyslipidemia, smoking status, baseline renal function (creatinine and urea), inflammatory markers (CRP and fibrinogen), and revascularization strategy (primary PCI, other PCI, or conservative management) were evaluated as potential confounders. Variables with a *p* value < 0.10 in univariable analyses and/or strong a priori clinical relevance were considered candidates for multivariable modeling. Because the composite inflammatory and nutritional indices (IPI, AISI, PNI, and CONUT) showed strong inter-correlations, multicollinearity was an important consideration during model construction. Inflammatory indices (IPI and AISI) were not entered into the final adjusted model due to substantial collinearity with CRP, albumin-based measures, and with each other. Among nutritional indices, both PNI and CONUT were evaluated; however, PNI remained the independent predictor, whereas CONUT was retained as a covariate because its inclusion did not introduce harmful multicollinearity. To construct a parsimonious multivariable Cox model, we followed a stepwise conceptual approach balancing clinical relevance and statistical stability. Only variables that contributed unique prognostic information and did not materially alter the effect estimates were retained, thereby minimizing overfitting while preserving key biological domains relevant to ACS prognosis. Accordingly, the final multivariable model included STEMI presentation (vs NSTEMI), age, Gensini score, CONUT score, and PNI. Results are presented as hazard ratios (HRs) with 95% confidence intervals (CIs). The proportional hazards assumption was evaluated and found to be acceptable. A two-sided *p* value < 0.05 was considered statistically significant.

## 3. Results

A total of 2400 participants were included in the study, comprising 800 control subjects, 800 patients with NSTEMI, and 800 patients with STEMI. Baseline demographic characteristics and laboratory findings are summarized in Table 2. Patients with NSTEMI and STEMI were significantly younger than the control group (*p* < 0.001 for both). Body mass index, systolic and diastolic blood pressure, and heart rate were significantly higher in both NSTEMI and STEMI groups compared with controls, with the highest values observed in the STEMI group (all *p* < 0.001). Markers of glycemic control, including fasting glucose and HbA1c levels, were progressively higher from controls to NSTEMI and STEMI patients (*p* < 0.001). Lipid parameters also differed significantly among groups, with higher total cholesterol, LDL cholesterol, and triglyceride levels observed in acute coronary syndrome groups compared with controls (*p* < 0.001). Cardiac biomarkers demonstrated marked elevations in NSTEMI and STEMI patients. Troponin I and CK-MB levels were significantly higher in the STEMI group compared with both NSTEMI patients and controls (*p* < 0.001). Inflammatory and renal parameters, including CRP, fibrinogen, urea, and creatinine, were also significantly increased in acute coronary syndrome patients, with the greatest elevations observed in the STEMI group (Table 2).

Hematological and nutritional indices are presented in Table 3. Hemoglobin levels, white blood cell count, and platelet counts were comparable among the three groups (all *p* > 0.05). In contrast, serum albumin levels were significantly higher in NSTEMI and STEMI patients compared with controls (*p* < 0.001). Total protein levels were also significantly higher in the STEMI group compared with NSTEMI patients (*p* < 0.01).

Inflammatory composite indices demonstrated substantial inter-correlations, with AISI showing a strong positive correlation with IPI (r ≈ 0.91, *p* < 0.001). Conversely, prognostic nutritional index (PNI) displayed a strong inverse correlation with CONUT score (r ≈ −0.70, *p* < 0.001), reflecting their opposing nutritional and inflammatory interpretations.

Composite scores and angiographic severity indices are shown in Table 4. The Gensini score was significantly higher in NSTEMI patients compared with controls and STEMI patients (*p* < 0.001), while STEMI patients also demonstrated higher scores than controls (*p* < 0.001). Both CONUT score and prognostic nutritional index (PNI) differed significantly among groups. NSTEMI and STEMI patients exhibited lower CONUT scores and higher PNI values compared with controls (*p* < 0.001). Survival time was significantly shorter in STEMI patients compared with NSTEMI patients (*p* < 0.001).

**Figure 1:**
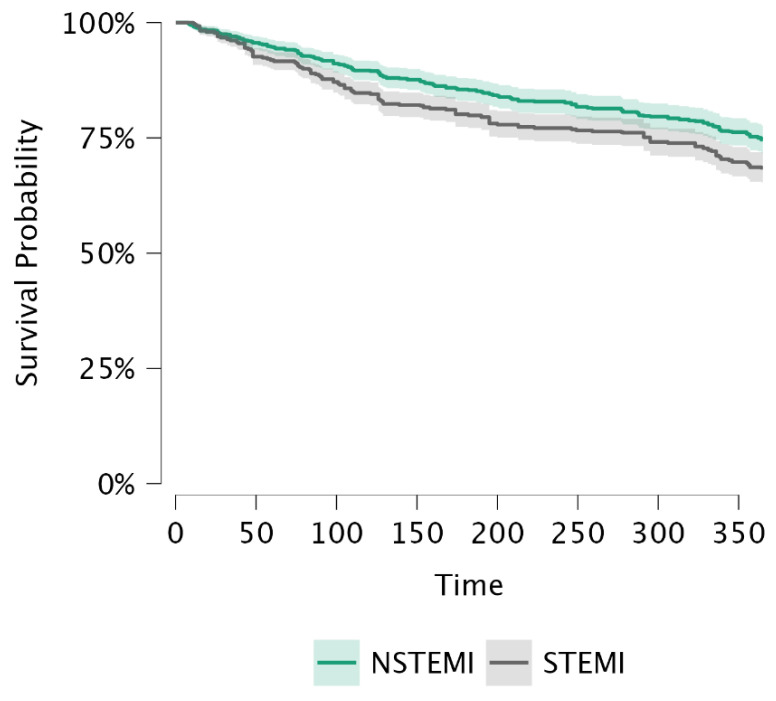
Kaplan–Meier survival curves comparing STEMI and NSTEMI patients. Kaplan–Meier survival analysis demonstrated significantly lower survival in patients with STEMI compared with NSTEMI (log-rank *p* = 0.005). Shaded areas represent 95% confidence intervals.

The mean follow-up duration was 307.47 ± 109.21 days in the overall ACS cohort, with NSTEMI patients followed for 315.54 ± 101.95 days and STEMI patients for 299.40 ± 115.51 days. Kaplan–Meier survival curves comparing STEMI and NSTEMI patients are shown in Figure 1. Survival was significantly lower in patients with STEMI compared with those with NSTEMI (log-rank *p* = 0.005). RMST was 315.5 days in the NSTEMI group and 299.4 days in the STEMI group (Table 5), indicating a shorter survival duration among STEMI patients during follow-up.

Additional univariable analyses showed that diabetes mellitus, BMI, baseline renal function, CRP, and revascularization strategy were not independently associated with mortality and did not change the effect estimates of STEMI, Gensini score, or PNI. Results of the multivariable Cox proportional hazards regression analysis for all-cause mortality are presented in Table 6. After adjustment for potential confounders, STEMI presentation was independently associated with an increased risk of mortality (HR 1.26, 95% CI 1.04–1.53, *p* = 0.018). Higher Gensini score was also identified as an independent predictor of mortality (HR 1.01 per point increase, 95% CI 1.00–1.02, *p* = 0.036). In contrast, higher PNI was independently associated with a reduced risk of mortality (HR 0.997, 95% CI 0.993–1.000, *p* = 0.045). Age and CONUT score were not independently associated with mortality in the multivariable model. Because the per-unit hazard ratio of PNI (0.997) represents a very small change, the effect was additionally expressed per 10-point increase. This scaling yields an HR of approximately 0.97, indicating about a 3% relative reduction in mortality for every 10-unit improvement in PNI.

## 4. Discussion

In this study, we comprehensively evaluated the clinical relevance of laboratory-derived systemic inflammatory and nutrition-related indices across the ACS spectrum (controls, NSTEMI, and STEMI) and identified several key findings. First, compared with controls, patients with ACS exhibited a higher cardiometabolic and inflammatory burden, including increased body mass index, blood pressure, and adverse glycemic markers, accompanied by elevations in systemic inflammatory and renal parameters (e.g., CRP, fibrinogen, urea, and creatinine). Cardiac injury biomarkers were markedly elevated in ACS and were most pronounced in STEMI, consistent with greater myocardial damage. Second, the composite indices demonstrated strong biological coherence, with a robust positive correlation between AISI and IPI and a strong inverse association between PNI and CONUT, supporting their complementary roles as markers of inflammatory load and immunonutritional balance. Third, angiographic disease severity differed significantly among ACS phenotypes, as reflected by the Gensini score, which was highest in NSTEMI. Finally, from a prognostic perspective, STEMI was associated with worse survival than NSTEMI, including shorter restricted mean survival time. In multivariable Cox analysis, STEMI presentation and higher Gensini score remained independent predictors of all-cause mortality, whereas higher PNI was independently protective; age and CONUT were not independently associated with mortality after adjustment. Collectively, these findings suggest that readily available inflammatory and nutrition-related indices may provide incremental value for risk stratification in ACS alongside conventional clinical assessment. The absence of an independent association between age and mortality in the multivariable model may be explained by several factors. First, STEMI presentation exerted a strong influence on short-term mortality in this cohort, likely overshadowing the contribution of chronological age. Second, the inclusion of the Gensini score, which captures anatomical burden with higher granularity, may have attenuated the effect of age once disease severity was accounted for. Finally, the relatively wide age distribution and limited follow-up period may have reduced the sensitivity for detecting age-related differences in mortality. Similar attenuation of age effects has been reported in ACS cohorts when more dominant clinical and angiographic predictors are included in multivariable models.

An unexpected observation in our study was that serum albumin levels and, consequently, PNI values were higher in ACS patients compared with controls. Although hypoalbuminemia is commonly associated with adverse cardiovascular outcomes, several factors may explain this apparent discrepancy. Blood samples in the ACS groups were obtained at hospital admission, prior to intravenous fluid administration. Acute sympathetic activation and early hemoconcentration during ischemic stress may transiently increase measured albumin concentrations. In contrast, the control group consisted of outpatient individuals undergoing cardiovascular evaluation, some of whom had chronic metabolic comorbidities that may be associated with low-grade inflammation and relatively lower albumin levels. Therefore, differences in clinical setting and volume status at the time of sampling may have influenced albumin distribution across groups. Importantly, despite these baseline differences, higher PNI remained independently protective in multivariable Cox analysis within the ACS cohort. This suggests that the prognostic significance of PNI reflects the integrated nutritional–inflammatory balance rather than isolated albumin levels alone and highlights the importance of interpreting albumin values within their acute clinical context.

In the current study, the observed pattern, namely younger ACS groups than controls; higher BMI, blood pressure, and heart rate; progressively worse glycemic indices; more atherogenic lipid profiles; greater troponin I and CK-MB elevations in STEMI; and higher CRP/fibrinogen with urea/creatinine in ACS, most pronounced in STEMI aligns with contemporary evidence suggesting that ACS severity reflects the combined impact of cardiometabolic burden (obesity, impaired glycemic control, and dyslipidemia), acute inflammatory activation, and early cardiorenal vulnerability. Admission hyperglycemia and stress hyperglycemia have repeatedly been linked to worse short- and long-term outcomes after MI, supporting the observation of progressively higher glucose/HbA1c across controls → NSTEMI → STEMI and the more adverse STEMI phenotype [22,23,24]. In parallel, higher CRP and fibrinogen levels in ACS are consistent with an amplified acute-phase response accompanying plaque rupture and myocardial injury; inflammation–nutrition composite approaches (e.g., hsCRP/albumin and fibrinogen/albumin ratios) have also been associated with mortality risk in ACS/STEMI cohorts, reinforcing the clinical relevance of capturing “inflammation + nutritional reserve” rather than single biomarkers alone [25,26,27]. The concomitant elevations in urea/creatinine among ACS patients are likewise compatible with evidence that baseline renal dysfunction and periprocedural acute kidney injury (AKI) in ACS particularly in STEMI portend worse prognosis, emphasizing the interconnected cardiorenal axis during acute ischemic events [28,29]. Finally, although our results indicate higher albumin in ACS versus controls, the broader literature shows that low albumin and malnutrition scores (e.g., CONUT) identify higher-risk coronary populations; differences in control group comorbidity mix, timing of sampling, or hydration/hemoconcentration at presentation may contribute to the directionality observed in the cohort while still supporting the overarching concept that nutritional–inflammatory balance is prognostically meaningful [27,28,29,30].

Although hemoglobin, total white blood cell count, and platelet counts were comparable across controls, NSTEMI, and STEMI in the present cohort, prior studies indicate that variations in these routine hematologic parameters particularly anemia and leukocytosis may still carry prognostic relevance in ACS, depending on case-mix, timing of sampling, and comorbidity burden. For instance, admission anemia and/or hospital-acquired anemia and higher WBC have been independently associated with increased in-hospital mortality in ACS cohorts, supporting the concept that oxygen-carrying capacity and inflammatory cell activation may modify early risk even when between-group differences are not apparent in all populations [31]. In contrast, serum albumin was higher in both NSTEMI and STEMI than in controls in our study, an observation that differs from much of the contemporary literature where lower albumin (as a negative acute-phase reactant and nutritional marker) has been linked to worse short-term outcomes and longer hospitalization in ACS/PCI settings [32]. This discrepancy may plausibly reflect differences in control group clinical characteristics (e.g., chronic inflammatory states or comorbidities lowering baseline albumin), as well as presentation-related hemoconcentration/relative hypovolemia, which can elevate measured serum protein fractions (including albumin and total protein), particularly in more intense clinical presentations such as STEMI [33]. Importantly, because PNI integrates albumin with immune status, its consistent association with outcomes across AMI/ACS cohorts supports interpreting nutritional–inflammatory balance using composite indices rather than isolated albumin levels alone, which may be sensitive to acute volume shifts [34].

Inflammatory composite indices in the current cohort showed strong internal consistency, with the very high correlation between AISI and IPI plausibly reflecting their shared biologic substrate and partially overlapping components (CBC-derived immune cell signals ± CRP/albumin), each intended to quantify the global inflammatory/thrombo-immune burden rather than a single pathway. In the contemporary AMI/ACS literature, higher AISI has been linked to worse in-hospital outcomes and cardiovascular death, supporting its role as an integrative marker of adverse inflammatory activation [35]. Likewise, higher IPI has been associated with angiography/PCI-related complications and adverse prognostic phenotypes (e.g., contrast-induced nephropathy risk after CAG/PCI and no-reflow during primary PCI in STEMI), reinforcing that these composite scores frequently track a similar high-risk inflammatory milieu, thereby making their tight correlation biologically expected [36]. In contrast, the strong inverse relationship between PNI and CONUT is consistent with their opposing construction and interpretation: PNI increases with better nutritional–immune reserve, whereas CONUT rises with worsening nutritional status (albumin- and cholesterol-related depletion with lymphopenia). In NSTEMI cohorts undergoing PCI, both indices have shown prognostic relevance in opposite directions (lower PNI and higher CONUT associated with higher long-term mortality), supporting the clinical meaning of this inverse coupling [37,38,39]. Regarding angiographic severity, the higher Gensini scores observed in NSTEMI compared with STEMI are concordant with reports that NSTEMI often reflects more diffuse/non-culprit coronary atherosclerotic burden and more multivessel disease, yielding higher (non-culprit) Gensini/SYNTAX measures than STEMI despite STEMI’s acute culprit occlusion [40]. Finally, the shorter survival in STEMI relative to NSTEMI in the present analysis aligns with the concept that STEMI can carry a greater early hazard related to infarct size and acute instability, although comparative outcomes across cohorts can vary by follow-up horizon and case-mix, with some studies reporting attenuated or non-significant differences after adjustment [41,42].

These survival analyses further underscore clinically meaningful differences between ACS phenotypes and highlight the complementary prognostic roles of anatomical disease burden and nutritional–inflammatory reserve. In the present cohort, STEMI patients had significantly lower survival than NSTEMI patients on Kaplan–Meier analysis, and the RMST difference (≈16 days) provides an intuitive absolute measure of time lost that can complement hazard-based estimates particularly when hazards may vary over follow-up [43]. Importantly, in the multivariable Cox model, STEMI presentation remained independently associated with mortality, consistent with large real-world datasets showing that STEMI is often linked to higher cause-specific mortality risk, although the STEMI–NSTEMI survival relationship may be context- and horizon-dependent (with some cohorts reporting higher longer-term mortality in NSTEMI driven by comorbidity burden) [44,45]. Beyond clinical phenotype, the finding that Gensini score independently predicted mortality supports the concept that angiographic atherosclerotic burden conveys incremental risk; contemporary evidence similarly associates higher Gensini scores with adverse outcomes after PCI and with short-term mortality among ACS populations [46,47,48,49]. Finally, the independent protective association of higher PNI aligns with growing evidence that PNI captures a clinically relevant nutritional–immune reserve and that lower PNI is consistently associated with higher mortality risk across CAD/AMI settings, reinforcing the value of integrating nutritional status into ACS risk assessment alongside anatomic severity and infarct presentation [50]. Although the hazard ratio for PNI appeared close to unity on a per-unit basis (HR 0.997), scaling the effect to a 10-point difference, a clinically more meaningful change, corresponds to an approximate 3% relative reduction in mortality risk (HR ≈ 0.97). Given the strong correlations among the composite indices, multicollinearity was expected. For this reason, inflammatory indices (IPI and AISI) were not included in the final multivariable model, as their components substantially overlapped with CRP, albumin, and other predictors. Among the nutritional indices, both PNI and CONUT were evaluated in multivariable analyses; however, PNI emerged as the independent predictor, whereas CONUT did not demonstrate an independent association but did not introduce harmful multicollinearity. This strategy reduced statistical collinearity while preserving the key biological domains relevant to ACS prognosis. Thus, the clinical relevance of PNI becomes clearer when interpreted over larger shifts in immunonutritional status rather than single-unit increments. This finding supports the role of PNI as a complementary prognostic marker rather than a standalone determinant of risk.

### 4.1. Strengths of the Study

This study leveraged a large cohort with equal group sizes (controls, NSTEMI, and STEMI), enabling comprehensive comparisons across the ACS spectrum. A key strength is the integrated evaluation of readily available systemic inflammatory and nutrition-related indices (IPI, AISI, PNI, CONUT) in conjunction with angiographic severity (Gensini score) and hard clinical outcomes (all-cause mortality). These analyses were supported by complementary survival approaches, including Kaplan–Meier curves and restricted mean survival time (RMST), as well as multivariable Cox modeling to identify independent prognostic associations.

### 4.2. Limitations of the Study

The present study has several limitations. First, its single-center, retrospective observational design limits causal inference and may reduce generalizability; residual confounding from unmeasured clinical and treatment-related factors cannot be excluded. Second, all inflammatory and nutritional indices were calculated from a single baseline blood sample; therefore, dynamic changes during hospitalization were not captured. Third, sampling occurred in different clinical contexts (acute presentation for ACS vs. routine evaluation for controls), and objective assessments of volume status or fluid balance were not systematically available; thus, potential hemoconcentration/hemodilution effects particularly relevant for albumin-based indices cannot be fully ruled out. Moreover, direct nutritional assessment (e.g., dietary intake, anthropometry, or validated malnutrition screening tools) was not available; therefore, “nutrition-related” indices should be interpreted as laboratory-based surrogates of immunonutritional status rather than comprehensive measures of nutritional status. In addition, strong inter-correlations among composite indices may introduce multicollinearity in multivariable models. Additionally, because GRACE and TIMI scores were not evaluated in parallel with the laboratory-derived indices, the degree to which these markers add prognostic information beyond established clinical risk models cannot be determined from this study. Finally, the lack of external validation and prespecified clinical cut-offs limits immediate clinical implementation and supports the need for prospective, multicenter studies to confirm these findings.

### 4.3. Clinical Application

Because IPI/AISI and PNI are derived from routine laboratory parameters, they can be readily implemented at admission to support early risk stratification in NSTEMI and STEMI. In practice, a high inflammatory burden (high IPI/AISI) and reduced nutritional–immune reserve (low PNI) could identify patients who may benefit from closer monitoring, intensified cardiometabolic optimization, and early multidisciplinary evaluation (including nutritional assessment) alongside angiographic severity assessment. Integrating these indices with coronary anatomy (Gensini) may help refine bedside decision-making by combining systemic vulnerability with disease burden to identify patients at highest short- and mid-term risk.

### 4.4. Future Directions

Prospective, multicenter studies should validate these findings in diverse ACS populations and standardize the calculation of IPI/AISI/PNI with predefined, clinically actionable cut-offs. Future work should assess the incremental value of these indices beyond established risk scores (e.g., GRACE/TIMI) using discrimination and reclassification metrics and should incorporate serial measurements to determine whether dynamic changes during hospitalization improve prognostication. Finally, interventional trials are needed to test whether index-guided strategies, particularly those targeting nutritional–inflammatory status, can translate into improved outcomes.

## 5. Conclusions

In this large cohort including controls, NSTEMI, and STEMI patients, ACS was associated with a greater cardiometabolic and systemic inflammatory burden, with the most pronounced myocardial injury and inflammatory disturbances observed in STEMI. Laboratory-derived inflammatory and nutrition-related indices demonstrated strong internal correlations, supporting their complementary interpretation as markers of inflammatory load and immunonutritional reserve. Angiographic severity differed across ACS phenotypes, and survival analyses consistently showed poorer outcomes in STEMI compared with NSTEMI. In multivariable models, STEMI presentation and higher Gensini score were independently associated with increased all-cause mortality, whereas higher PNI was independently protective. These findings suggest that readily available inflammatory and nutrition-related indices may provide incremental prognostic information alongside conventional clinical and angiographic assessment. Prospective, multicenter studies are needed to validate these observations and define clinically actionable thresholds.

## Figures and Tables

**Table 1:** Assessment of nutritional status with CONUT.

CONUT Score	Level of Malnutrition			
	Normal	Mild	Moderate	Severe
Serum Albumin (g/dL)	≥3.5	3–3.4	2.5–2.9	<2.5
Score	(0)	(2)	(4)	
Total Lymphocytes (×10^9^/L)	≥1600	1200–1599	800–1199	<800
Score	(0)	(1)	(2)	(3)
Total Cholesterol (mg/dL)	≥180	140–179	100–139	<100
Score	(0)	(1)	(2)	(3)
Total Score	(0–1)	(2–4)	(5–8)	(9–12)

**Table 2:** Demographic characteristics and laboratory findings.

Parameter	Control (n = 800)	NSTEMI (n = 800)	STEMI (n = 800)
Age (years)	64.40 ± 16.11	61.23 ± 15.25 a ***	62.35 ± 16.66 a *
BMI (kg/m^2^)	24.49 ± 2.65	25.78 ± 4.11 a ***	27.39 ± 1.92 a ***, b ***
SBP (mmHg)	121.9 ± 13.5	134.3 ± 17.7 a ***	131.8 ± 14.9 a ***
DBP (mmHg)	69.2 ± 9.0	82.2 ± 5.5 a ***	75.4 ± 7.7 a ***, b ***
Heart rate (bpm)	75.3 ± 6.2	76.7 ± 8.3 a ***	78.3 ± 9.5 a ***, b ***
Fasting glucose (mg/dL)	92.2 ± 5.7	98.0 ± 22.1	126.8 ± 38.5 a ***, b ***
HbA1c (%)	5.24 ± 0.54	5.68 ± 0.67 a ***	7.51 ± 1.77 a ***, b ***
Total cholesterol (mg/dL)	149.4 ± 24.6	186.2 ± 49.0 a ***	162.3 ± 34.8 a ***, b ***
LDL (mg/dL)	85.8 ± 20.0	127.7 ± 102.4 a ***	97.4 ± 27.7 a ***, b ***
HDL (mg/dL)	38.9 ± 9.4	48.9 ± 14.2 a ***	38.4 ± 9.3 b ***
Triglycerides (mg/dL)	94.0 ± 22.4	168.8 ± 147.9 a ***	127.0 ± 36.8 a ***
Troponin I (ng/mL)	0.06 ± 0.02	1.81 ± 1.76 a ***	33.15 ± 13.38 a ***, b ***
CK-MB (ng/mL)	13.1 ± 4.1	62.2 ± 33.8 a ***	349.2 ± 258.6 a ***, b ***
CRP (mg/L)	3.20 ± 1.59	27.63 ± 33.32 a ***	21.77 ± 26.99 a ***, b ***
Fibrinogen (g/L)	4.01 ± 1.26	4.57 ± 1.33 a ***	4.74 ± 1.25 a ***
Urea (mg/dL)	34.3 ± 9.6	55.0 ± 26.4 a ***	42.4 ± 22.1 a ***, b ***
Creatinine (mg/dL)	0.90 ± 0.21	1.06 ± 0.49 a ***	1.04 ± 0.67 a ***

Values are presented as mean ± standard deviation. Post hoc comparisons were performed using Tukey HSD test; *p* < 0.05 was considered statistically significant. a represents vs. control, b represents vs. NSTEMI; *: *p* < 0.05, ***: *p* < 0.001.

**Table 3:** Hematological and nutritional indices.

Parameter	Control (n = 800)	NSTEMI (n = 800)	STEMI (n = 800)
Hemoglobin (g/dL)	14.04 ± 1.73	14.03 ± 1.75	14.01 ± 1.77
WBC (×10^3^/µL)	7.43 ± 2.04	7.37 ± 1.96	7.37 ± 1.98
Platelets (×10^3^/µL)	298.7 ± 87.4	298.5 ± 85.1	300.1 ± 82.8
Albumin (g/dL)	3.79 ± 0.34	3.94 ± 0.47 a ***	3.96 ± 0.29 a ***
Total protein (g/dL)	6.76 ± 0.70	6.73 ± 0.83	6.84 ± 0.60 b **

Values are presented as mean ± standard deviation. Post hoc comparisons were performed using Tukey HSD test; *p* < 0.05 was considered statistically significant. a represents vs. control, b represents vs. NSTEMI; **: *p* < 0.01, ***: *p* < 0.001.

**Table 4:** Composite scores and angiographic severity.

Parameter	Control (n = 800)	NSTEMI (n = 800)	STEMI (n = 800)
Gensini score	42.7 ± 11.8	50.2 ± 14.3 a ***	45.1 ± 12.4 a ***, b ***
CONUT score	1.66 ± 1.28	1.12 ± 1.30 a ***	1.16 ± 1.11 a ***, b *
PNI	187.3 ± 23.9	225.6 ± 50.0 a ***	201.9 ± 34.1 a ***, b ***
Survival time (days)	0	315.5 ± 102.0	299.4 ± 115.5 b ***

Values are presented as mean ± standard deviation. Post hoc comparisons were performed using Tukey HSD test; *p* < 0.05 was considered statistically significant. a represents vs. control, b represents vs. NSTEMI; *: *p* < 0.05, ***: *p* < 0.001.

**Table 5:** Kaplan–Meier survival analysis summary in STEMI and NSTEMI patients.

Strata	N	Events	Restricted Mean	Standard Error	Chi-Square	df	*p*
NSTEMI	800	203	315.540	3.602	7.953	1	0.005
STEMI	800	252	299.401	4.081			

Values are presented as restricted mean survival time (RMST). Survival curves were compared using the log-rank test.

**Table 6:** Multivariable Cox proportional hazards regression analysis for all-cause mortality.

Variable	Hazard Ratio (HR)	95% CI	*p* Value
STEMI (vs. NSTEMI)	1.26	1.04–1.53	0.018
Age (per years)	1.00	0.99–1.01	0.635
Gensini score (per point)	1.01	1.00–1.02	0.036
CONUT score (per point)	0.97	0.88–1.07	0.590
PNI (per unit)	0.997	0.993–1.000	0.045

STEMI indicates ST-elevation myocardial infarction; NSTEMI, non-ST-elevation myocardial infarction; PNI, prognostic nutritional index; CI, confidence interval. Note: Hazard ratios represent the effect per one-unit increase in continuous variables. For PNI, a 10-unit increase corresponds to an approximate 3% relative reduction in mortality risk (HR ≈ 0.97).

## Data Availability

The data underlying this article are available in the article. If needed, please contact the corresponding author. The email address is nedimuzun@gmail.com.
